# *EMT en Español Para Autismo*: A Collaborative Communication Intervention Approach and Single Case Design Pilot Study

**DOI:** 10.1007/s10803-024-06322-5

**Published:** 2024-04-13

**Authors:** Natalie S. Pak, Tatiana Nogueira Peredo, Ana Paula Madero Ucero, Ann P. Kaiser

**Affiliations:** 1 Department of Special Education, Peabody College, Vanderbilt University, Nashville, TN USA; 2 Present Address: Department of Communication Sciences and Disorders, University of South Florida, Tampa, FL USA

**Keywords:** Early language intervention, Single case, Spanish, Autism

## Abstract

The primary purpose of the current pilot study was to test the effects of an adapted and collaborative intervention model with a systematic teaching approach on Latina Spanish-speaking caregivers’ use of *EMT en Español Para Autismo* strategies with their young children on the autism spectrum. A multiple baseline across behaviors single case design was replicated across two dyads. A series of family interviews and a direct therapist-child intervention phase supported individualization of the intervention. Families were provided speech generating devices as part of their children’s intervention protocol. Caregivers were taught to use *EMT en Español Para Autismo* strategies with aided language input. Strategies included contingent target-level and proximal target-level language modeling, linguistic expansions, and communication elicitations. Secondary variables measured included generalization of strategy use to unsupported interactions and at a 2-month follow-up, child communication outcomes, and social validity. There was a strong functional relation for one dyad between the adapted and collaborative intervention and caregiver use of EMT strategies. The functional relation was weakened by behavioral covariation for the other dyad. Children increased the quantity and diversity of their communication during the study. Caregivers generalized their use of most EMT strategies and reported most aspects of the approach to be socially valid. The current study provides an initial demonstration of an effective model for adaptation and individualization of naturalistic developmental behavioral interventions for Latino Spanish-speaking families with children on the autism spectrum.

Early diagnoses of autism are increasingly prevalent in the United States, affecting an estimated 1 in 46 preschool-aged children across all races and ethnicities and 1 in 34 Hispanic children (Shaw et al., 2023). Latino Spanish-speaking (LSS) families face multiple systemic barriers to accessing early intervention for their young children on the autism spectrum and are more likely than non-Latino White children to receive no or inadequate services (Stahmer et al., 2019; Zuckerman et al., 2017). Caregiver-mediated (or implemented) interventions have been shown to positively influence children’s language outcomes for monolingual English-speaking children (Heidlage et al., 2020; Roberts et al., 2019). In this article, caregiver refers to children’s primary caregivers in the home (e.g., parent, other family member). Importantly, LSS caregivers report a desire to be partners in the delivery of intervention for their children on the autism spectrum, and they report children’s communication skills to be a high priority for intervention (DuBay et al., 2018). In a scoping review of the literature, DuBay (2022) identified 19 studies investigating culturally adapted caregiver-mediated interventions for Latino families and children on the autism spectrum. Only two involved interventions specifically targeting children’s early communication skills (Gevarter et al., 2022; Meadan et al., 2020). To reduce the disparities in early intervention services, more culturally and linguistically adapted caregiver-implemented language interventions for children on the autism spectrum are necessary (Martinez-Torres et al., 2021).

## EMT en Español

*EMT en Español* is a Spanish language, caregiver-mediated adaptation of Enhanced Milieu Teaching (EMT) that has been tested with LSS families and their preschool children with language delays (Peredo et al., 2018, 2022). *EMT en Español* and EMT are naturalistic developmental behavioral interventions (NDBIs) which involve use of behavioral principles to teach developmentally appropriate communication skills in naturalistic settings (Schreibman et al., 2015). Among NDBIs, EMT is uniquely focused on improving child language and communication development and has been demonstrated to be effective for children with a variety of etiologies of language impairments (Kaiser & Hampton, 2017; Kaiser et al., 2021; Roberts & Kaiser, 2015; Wright et al., 2013).

Cultural and linguistic adaptations to interventions such as EMT may be linked to dimensions of the ecological validity model (EVM), a framework designed specifically for adapting interventions to be more culturally sensitive for Spanish-speaking families (Bernal et al., 1995). According to this model, there are eight dimensions that can influence the cultural consistency of an intervention for a given client or community. These dimensions are language, persons, metaphors, content, concepts, goals, methods, and context. Adaptations to *EMT en Español* have addressed several dimensions of the EVM (see Peredo et al., 2018, and Peredo et al., 2022, for more details). For example, rather than simply following the child’s lead, caregivers are coached to first comment on the child’s focus of interest within *adult-directed* activities. This addresses the dimensions of content and concepts. Additional adaptations have been implemented in the procedures and delivery of intervention (method). For example, interventionists speak Spanish with families (language, persons) and deliver intervention in homes during familiar and/or valued routines (context, goals) (Peredo et al., 2018, 2022).

These adaptations have been tested in two studies. Using a single-case experimental design, Peredo et al. (2018) demonstrated that three Spanish-speaking mothers from Mexico applied *EMT en Español* strategies with their preschool children with developmental language disorders when the mothers were taught using a systematic training approach (Teach-Model-Coach-Review or TMCR; Roberts & Kaiser, 2015). The mothers generalized use of most *EMT en Español* strategies to a novel context at home and reported using the strategies additional times throughout the week. Results for LSS caregivers receiving systematic instruction to use *EMT en Español* were also positive in a small randomized trial (Peredo et al., 2022). Twenty LSS caregivers and their children with language delays (age range 29–43 months) were randomized to a 24-session intervention at home (*n* = 10) or waitlist control group (*n* = 10). There were statistically significant intervention effects for caregivers’ use of matched turns, expansions, and linguistic targets (*d* = 1.24–1.90).

## EMT en Español Para Autismo

The current study was a pilot investigation of *EMT en Español Para Autismo*, an adaptation of *EMT en Español* aiming to address the specific needs of LSS families of children on the autism spectrum. Prior to the study, four LSS primary caregivers of children on the autism spectrum provided feedback on *EMT en Español* materials in a focus group format. The focus group caregivers were positive about the materials, reported the materials were relevant to them, and noted areas in which they would benefit from more information. This feedback was combined with clinical expertise and experience from previous EMT studies with children on the autism spectrum (e.g., Hampton et al., 2021) to make adaptations for the current study.

The first adaptation was to include information to expand caregivers’ knowledge about autism. Focus group findings were consistent with reports that LSS parents of children on the autism spectrum often begin evaluation and treatment services with limited knowledge about autism, which can lead to self-blame for their children’s challenges (Chlebowski et al., 2018; Zuckerman et al., 2017). The second adaptation was to teach caregivers *individualized* strategies for promoting child engagement in interactions and activities, which was a need reported by focus group caregivers. Strategies to support children’s engagement have been reported in previous EMT and *EMT en Español* studies. These include: (a) arranging the setting to support children’s contact with activities and to minimize distractions, (b) choosing high interest toys, (c) sitting at the child’s level, (d) scaffolding play and engagement, (e) shifting activities when children lose interest, and (f) specific behavior supports such as use of timers and first-then charts (Hampton et al., 2019, 2021; Peredo et al., 2018, 2022). In the current pilot study, many of the same strategies were employed; however, the selected strategies were individualized based on family concerns and preferences expressed throughout the study and based on an initial phase of therapist-delivered child intervention. The third adaptation was to provide access to high-tech augmentative and alternative communication (AAC) for children who began the study with little to no expressive spoken language. AAC, which includes various modes of communication used instead of or in addition to speech, may be important for young children at high risk of delayed development of spoken language (Beukelman & Light, 2020). Specifically, children received speech-generating devices (SGDs) in the form of iPad minis with the Proloquo2Go communication app (AssistiveWare, 2023). Spanish and English were both available on the Proloquo2Go app; Spanish vocabulary was primarily used during the study for language modeling, with vocabulary selections made collaboratively with each family. Families were coached to model language with both the SGD and speech while delivering *EMT en Español Para Autismo* with their children (i.e., aided AAC modeling; Beukelman & Light, 2020).

The primary purpose of this pilot study was to assess the effects using a systematic teaching approach to teach LSS caregivers of children on the autism spectrum to implement *EMT en Español Para Autismo*. We posed the following research questions: (a) Do LSS caregivers of children on the autism spectrum use *EMT en Español Para Autismo* strategies during *coached* caregiver-child interactions when taught using the TMCR approach? (b) Do LSS caregivers use *EMT en Español Para Autismo* strategies during caregiver-child interactions *without* coaching during and after the intervention period when taught using the TMCR approach? (c) Do LSS children on the autism spectrum increase the frequency and diversity of their communication when their caregivers are taught *EMT en Español Para Autismo* strategies? (d) How do caregivers perceive the intervention approach?

## Method

### Experimental Design

**Fig. 1:**
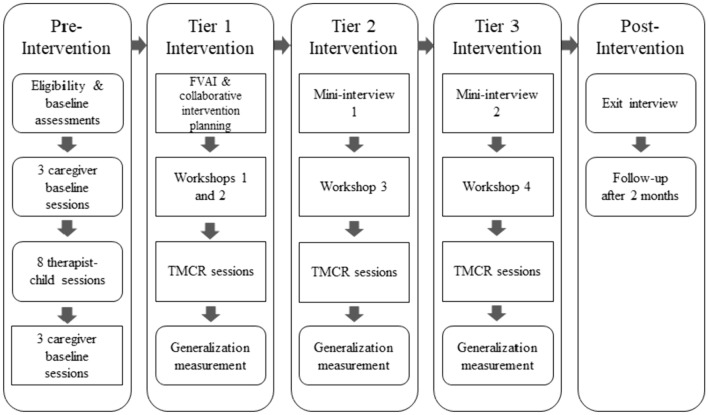
Flowchart of study activities. The order of phases is pre-intervention, Tier 1 intervention, Tier 2 intervention, Tier 3 intervention, and post-intervention. Within each intervention tier, there is an interview activity, workshop, TMCR intervention, and a generalization session. Boxes with square corners indicate activities that were part of the experimental design

The experimental design was a single-case multiple baseline design across behaviors replicated across caregiver-child dyads (Baer et al., 1968). In multiple baseline designs across behaviors, participants are taught functionally similar but independent behavior sets with a time-lagged introduction of intervention for each behavior set (Gast et al., 2018). In the current study, the behavior sets (i.e., tiers of the intervention) were sets of *EMT en Español Para Autismo* strategies: (a) contingent target-level language modeling, (b) contingent higher-level language modeling including proximal targets and expansions, and (c) communication elicitation strategies (see Table 1 for definitions). Environmental arrangement strategies to support child engagement and communication (e.g., eliminating distractions, using timers if needed to increase duration of child play, reducing questions and instructions) were taught in Tier 1 along with target level language models. The sequence of study phases and activities is shown in Fig. 1.

**Table 1:** Dependent variables definitions

Caregiver variable	Definition
Target-level language models (Tier 1)	The number of times during the 10-min coding period the caregiver used target language level utterance following a child’s communicative turn within 3 s (matched turn), following their own matched turn that was directly related in content (related turn), or following 3 s in which the child did not take a communicative turn (extra turn). Simultaneously model using AAC and verbal language Target level utterances included: Singular article + noun (e.g., una paleta/a popsicle) Inflected common verbs in present tense/present progressive (e.g., están jugando/they are playing) Modifier (e.g., caliente/hot or está caliente/it’s hot) Location words (e.g., adentro/inside)
Proximal target-level language models (Tier 2)	Identical to above, except that the utterances were proximal target level Proximal target level utterances included: Plural article + noun (e.g., las burbujas/the bubbles) Article + noun + present tense/present progressive verb (e.g., los niños están jugando/the children are playing) Article + noun + modifier (e.g., la torre grande/the big tower) Reflexive verb (e.g., se acabó/it’s finished) Pronoun (or implied pronoun) + preterit or other verb tense (e.g., caminaba/it was walking) Verb + object (direct or indirect, attached or unattached) (e.g., la comió/he ate it; lavamos los platos/we wash the dishes) Negation + verb (e.g., no tenemos nada/we don’t have anything)
Expansions (Tier 2)	Aggregate number of the following: The number of times the caregiver responded to child utterances without changing the child’s communicative intent by: (a) Adding 1–3 words to the utterance (b) Recasting the child’s semantically incorrect or nonspecific (e.g., esto [this]) word (c) Recasting the child’s grammatically incorrect word or utterance
Communication elicitations (Tier 3)	Time Delays: Least-to-most prompting sequences that include *nonverbal* cues to elicit requesting at the child’s target language level. May include creating situations in which the child needs assistance, presenting two choices (e.g., holding up two objects the child is likely to want), or pausing within a routine Milieu Prompting Episodes: Least-to-most prompting sequences that include *verbal* cues to elicit requesting at the child’s target language level. Verbal cues may be open questions (“¿Qué quieres?”/What do you want?), choice questions (“¿Quieres ___ o ___?”/Do you want ___ or ___?), or model prompts (“Di ___”/Say ___) Questions (asked during book-reading): question sequences that the adult asks during shared book-reading for which the expected response is at the target language level. Questions might be “¿Qué es?” (What is it?) or “¿Qué están haciendo?” (What are they doing?). If the child does not respond correctly, the adult models the correct response and repeats the question up to 2 times. If the child responds correctly, the adult responds with a linguistic expansion

Child Variable	Definition
Number of Total Words (NTW)	Number of total words (spoken or AAC) the child used during the 10-min session
Number of Different Words (NDW)	Number of different words (spoken or AAC) the child used during the 10-min session
Frequency of Social Communication	Number of utterances or acts in which the child used spontaneous and elicited words (spoken or AAC), communicative vocalizations, or communicative gestures during the 10-min session. To be considered communicative, gestures such as reaches had to be accompanied by a vocalization or eye contact

When the child activated symbols using AAC that contained multiple words (e.g., “ya terminé”/all done, “me gusta”/I like it), these single-symbol phrases were transcribed as single words using an underscore (e.g., me_gusta)

The study phases were (a) pre-intervention, (b) teaching caregivers three sets of EMT strategies using the TMCR approach across tiers of the intervention design, and (c) post-intervention assessment. The pre-intervention phase included three initial baseline sessions with the caregiver, eight sessions of direct therapist delivery of the intervention to the child, and a second set of three baseline sessions. Measuring caregiver baseline performance prior to and after therapist-child intervention was included to detect any change in caregiver use of strategies from watching the therapist use the strategies before the TMCR intervention. The experimental design was implemented in the second set of baseline sessions and the planning, teaching, and coaching components of the intervention during the TMCR phase (boxes with square corners in Fig. 1). The post-intervention phase included a caregiver exit interview immediately after intervention and a follow-up observation 2 months later.

### Recruitment

Caregiver-child dyads who met the following criteria and wished to participate in the study were recruited: (a) Spanish was the primary language spoken in the home; (b) the child had an autism diagnosis or flagged on an autism screening measure; (c) the child was 30–42 months old at the beginning of intervention; (d) the child had a Total Language Score at least 1.5 *SD* below the mean standardized score on the *Preschool Language Scales, 5th edition Spanish* (PLS-5 Spanish; Zimmerman et al., 2012); and (e) at least one primary caregiver was willing and able to participate in the intensive intervention for several months. Participants were recruited from a list of children who were assessed for eligibility for an ongoing randomized controlled trial (Kaiser & Peredo, 2019–2024) but were excluded because the children already had an autism diagnosis or exhibited characteristics of autism based on the *Screening Tool for Autism in Toddlers and Young Children* (STAT; Stone & Ousley, 2008). A bilingual member of the research team called participants who had consented to being contacted for future studies for a phone screening. Subsequent in-person eligibility assessments were conducted in families’ homes. Interested families whose children demonstrated characteristics of autism based on the STAT but did not yet have a diagnosis were provided with a full evaluation including administration of the TELE-ASD-PEDS (Corona et al., 2020) and a diagnostic interview by qualified providers. Prior to any study activities, consent was obtained from caregivers indicating that they wished to participate and that they gave consent for their children to participate. Written consent forms and verbal explanations of the consent forms were in Spanish. All study procedures and materials were approved by a university Institutional Review Board (IRB). Participating families received toys and books (shape sorter, blocks, bubbles, and two bilingual picture books) at the beginning of the study valued at approximately $50. Additional incentives included intervention materials that were collaboratively selected with the family during the individualization process described in the following section.

### Intervention Planning and Individualization

Individualizing the intervention at the beginning of the study occurred during the therapist-child intervention phase and the series of interviews (see Fig. 1). The primary purposes of the therapist-child intervention phase were to (a) give the child experience in the intervention context as a foundation for the caregiver-implemented intervention and (b) provide the research team with specific information about how to best individualize intervention based on their interactions with the child. Family members in addition to the participating caregiver were invited to the initial interview and planning session, which occurred after all baseline sessions were completed and prior to any caregiver instruction (see Fig. 1). The Family Values and Activities Interview (FVAI) was administered in Spanish by the interventionist using the FVAI protocol (Peredo, 2016). The first part of this semi-structured protocol was a series of open-ended questions about the family values, goals, and beliefs about communication. The second part included questions about the activities that occurred frequently, were important to the family, or both.

During the planning portion of the session, the family and interventionist first selected specific routines or activities that were typical for each family and could be used in the intervention sessions to practice the *EMT en Español Para Autismo* intervention strategies with coaching. Second, the interviewer, interventionist, and family collaborated to select additional play materials (within a $50 budget per family) that would be engaging for the child and facilitate communicative interactions. Third, families and therapists determined whether to introduce the SGD if the child used fewer than five spoken words at the beginning of intervention and during therapist-child intervention sessions. When applicable, families were provided iPad minis loaded with Proloquo2Go. Activity grid displays with Spanish vocabulary were primarily used for this pilot study. The families kept the SGDs between sessions during the study and after the study ended. Prior to beginning the caregiver-implemented intervention phase, children’s abilities to visually scan symbols on the iPad were tested using a “chase the ball” task to determine the grid size (see Hampton et al., 2020, for a description). Core vocabulary words (e.g., sí/yes, no, poner/to put) were added to each page, and activity pages were individualized to the participant. Symbols were added on an ongoing basis based on caregiver preferences and therapist suggestions, ensuring that an adequate number of verbs, nouns, and adjectives were available, and that vocabulary matched the family’s dialect and vocabulary preferences (Bernal et al., 1995; Binger et al., 2023).

Each family also participated in two shorter mid-intervention interviews (“mini-interviews”; see Fig. 1 and Online Resource) with the interventionist. The mini-interviews occurred immediately before the introduction of Tier 2 and Tier 3 strategies. During mini-interviews, the interventionist asked the families how they felt about the intervention, their child’s progress, and any changes in family activities relevant to intervention.

### Participants

Five dyads completed in-person screening for the study. One dyad did not enroll in the study due to limited ability to participate in study sessions multiple times per week. Two dyads enrolled in the study but dropped out before starting intervention or before completing Tier 1 of intervention. In both cases, the caregivers did not wish to continue with the study sessions because their children became eligible to start receiving services at school or from other providers. Table 2 shows characteristics of the two dyads who enrolled and completed the study.

**Table 2:** Participant characteristics

Participant characteristics	Dyad 1	Dyad 2
Age of child at baseline	33 months	31 months
Gender of child	Boy	Boy
Number of different spoken words^a^	1	0
PLS-5 Spanish total language score	50	55
Participating caregiver relationship to child	Grandmother	Mother
Age of participating caregiver	51 years	40 years
Family country(s) of origin	Mexico, Cuba	Venezuela
Participating caregiver length of time in US	2 years	4 years

PLS-5 Spanish Preschool Language Scales, 5th edition Spanish (Zimmerman et al., 2012)

^a^In two 20-min language samples (one in English, one in Spanish)

Dyad 1 included a 33-month-old boy and his maternal grandmother, referred to as Daniel and Dayana. Daniel received an autism diagnosis from an evaluation team in Mexico during the study prior to the FVAI and planning session. Daniel was not receiving any additional services at the beginning of the study, but he began attending full-day monolingual English-speaking preschool during Tier 2 of the study intervention. Dayana and Daniel’s mother participated in the initial FVAI and planning session. Per the family’s report and observation during therapist-child sessions, Daniel enjoyed playing with a variety of toys, movement (e.g., jumping on a trampoline), and looking at books. He communicated primarily by vocalizing, leading others by the hand, and giving objects. The therapist and family decided to introduce the SGD, which was available during all subsequent TMCR and generalization sessions except for one session when the battery had died. Although Daniel preferred reading books independently and would turn away when others joined him, shared book-reading was valued by the family and was incorporated into TMCR sessions. The additional materials collaboratively selected for intervention included toys representing various foods and cooking tools, board books, and a pop-up toy. Snack and mealtime routines were preferred activities for Daniel and were selected as contexts for caregiver practice and coaching. Daniel’s mother, father, and grandmother all participated in the first mini-interview (prior to Tier 2) and the exit interview. Only Dayana participated in the second mini-interview (prior to Tier 3). During mini-interviews, the family discussed child progress that they noticed, such as that he was making eye contact more often and sleeping better. After the first mini-interview, drawing with markers was added as an intervention session activity and handouts were provided to help with ongoing potty training outside of sessions. Although shared book-reading continued to be a struggle, the family continued to state its importance and it remained an intervention session activity.

Dyad 2 included a 31-month-old boy and his mother, referred to as Luis and María. Luis demonstrated signs of autism during screening and was subsequently diagnosed during a professional evaluation arranged by the research team. Luis attended a bilingual English- and Spanish-speaking childcare for approximately 4–7 h each weekday at the beginning of the study, but his enrollment was inconsistent during the study. Each week, he received occupational therapy 30 min and speech-language therapy 60 min in English. His mother had monthly telepractice consultations in Spanish regarding strategies to support Luis at home. María participated in the FVAI and planning session. Per caregiver report and observation during therapist-child sessions, Luis enjoyed taking walks, watching television, shared book reading, blocks, tickles, and sensory play (e.g., Play-Doh). He communicated by vocalizing, using gestures such as reaching and giving, and a few spoken words (e.g., mamá, no). María was hesitant about the SGD, as she wanted to limit her children’s screen time; however, she agreed to try using it for a few sessions before deciding. In the fifth TMCR session with the SGD, María mentioned that she liked that he was trying to use the device more frequently to communicate. The SGD was available in all subsequent TMCR and generalization sessions. The additional materials selected for intervention included puppets, books, and a Play-Doh set. Preparing and eating food, getting dressed, and combing hair were preferred routines for Luis; these were incorporated into TMCR sessions as routines for practice and coaching with *EMT en Español Para Autismo* strategies. María and Luis’s grandmother participated in mini-interviews. They reported noticing changes in the child’s communication and behavior, including more vocalizations and pointing, more interest in play, and more awareness of his surroundings. They also shared that they still hoped he would talk more. Brushing teeth and washing dishes were routines added to TMCR sessions based on feedback during mini-interviews. Playing with Play-Doh became a favorite activity for Luis.

### Procedures

Sessions occurred up to three times per week (approximately 120–180 min/week) in families’ homes and were video recorded. One interview with Dayana occurred via a Zoom (version 5.13.7) videoconference due to family illness. There were two primary interventionists, one for each of the two participating families. The first interventionist (female, 31 years old, Korean/White) was a doctoral candidate in Special Education and a speech-language pathologist with 4 years of training and experience delivering EMT and *EMT en Español* to young children with language delays in research settings. She was a proficient Spanish speaker, a native English speaker, and a lifelong resident of the United States. The second interventionist (female, 42 years old, Latina) had over 20 years of clinical experience in language and behavioral interventions with young children. She had a master’s degree in psychology and over 5 years of experience with *EMT en Español* and TMCR in research settings. She was a native Spanish speaker, a fluent English speaker, and had been a resident of the United States (9 years) and Mexico.

#### Pre-Intervention Phase

Pre-intervention activities are shown in Fig. 1. During caregiver baseline sessions (approximately 25 min per visit), the therapist video recorded the caregiver and child interacting in typical play or book-reading contexts for 15 min. Families were provided with the standard toys and books at the first session. During therapist-delivered intervention sessions (approximately 35 min per visit), the intervention lasted 25 min, including 20 min of play with toys and 5 min of book reading. The caregiver was invited but not required to observe the session. No caregiver instruction occurred in this phase.

#### Teach-Model-Coach-Review Phase

TMCR sessions lasted approximately 1 h and contained four segments corresponding to teach, model, coach, and review. The duration and activities of each are shown in Table 3.

**Table 3:** Teach-model-coach-review session structure

Component	Duration	Activities
Teach (introduction of new strategy)	20–30 min	Using PowerPoint slides on a laptop, the interventionist: 1. Explained the specific *EMT en Español Para Autismo* strategy 2. Provided a rationale for its use in supporting the child’s participation and communication 3. Related the strategy use to their child’s skills and needs 4. Showed video examples of the strategy being used 5. Discussed examples and answered caregiver questions
Teach (all other sessions)	5–10 min	1. Recap of the strategy 2. Rationale for using the strategy 3. Active learning around the strategy (hypothetical scenarios, video examples, or intervention planning)
Model	10 min	1. Interventionist guided caregiver to watch for target strategies (e.g., “note the different specific words I use during this activity”) 2. Interventionist delivered *EMT en Español Para Autismo* with the child 3. Asked caregiver what they noticed about use of the target strategies and the child’s responses
Coach	15 min	1. Caregiver used strategies during play (10 min), book-reading (2–3 min), and routines (2–3 min) with their child 2. Interventionist provided in the moment general positive feedback, specific feedback, and suggestions 3. Interventionist supported environmental arrangement through support with materials management and SGD troubleshooting
Review	5–10 min	1. Interventionist asked caregiver how the session felt that day and follow-up questions 2. Interventionist answered caregiver questions 3. Discussion of how the caregiver could practice the strategies independently before the next session

The Teach portion included a workshop (20–30 min) when a new strategy was introduced (i.e., at the beginning of the phase change for each tier), and the remaining sessions included a shorter review of the target strategies (5–10 min). During the Model portion (10 min), the therapist modeled all *EMT en Español Para Autismo* strategies with the child, including those that had not yet been taught to the caregiver. To avoid behavioral covariation across tiers (Gast et al., 2018), the therapist narrated and discussed her use of only the strategies that had been introduced to the caregiver. In the Coach segment (15 min), the caregiver used strategies during play, book-reading, and routines that had been collaboratively selected during the planning meeting. The interventionist coached the caregiver and provided brief positive feedback to support her use of the targeted strategies. The interventionist modeled and coached the caregiver to model spoken language targets while simultaneously activating corresponding symbols with the SGD (Biggs et al., 2018; Sevcik et al., 1995). In some cases, 10 min of play was divided into shorter segments with visual timers for the child. Finally, in the Review segment (5–10 min), the caregiver and therapist reviewed and reflected on the session. Overall, the child received intervention from the caregiver for 15 min during the Coach component and from the interventionist for 10 min during the Model component. Only 10 min of caregiver-child interaction were coded, as described below.

Generalization sessions lasted 15 min and occurred four times for each family during the TMCR phase—once before each of the three workshops, and once before the exit interview. Like baseline sessions, the therapist did not provide any coaching or instruction before, during, or after the caregiver-child interaction. Like TMCR sessions, the therapist asked the family to engage in the three activity contexts: play (10 min), book-reading (2–3 min), and routine (2–3 min) (Table 3).

#### Post-Intervention Phase

Daniel’s mother, father, and grandmother participated in the exit interview (English version available in Online Resource). María and Luis’s grandmother participated in the exit interview. The exit interviews were conducted in Spanish by the interventionist who did not coach the family. Questions were related to the utility of *EMT en Español Para Autismo* strategies, approximately how often the caregivers practiced the strategies each week during different types of activities, and how the intervention could be improved for families who would participate in the future. The interviewer also asked families to rate the effectiveness and appropriateness of each of the *EMT en Español Para Autismo* strategies on a 5-point Likert-type scale (1 = ineffective or inappropriate, 5 = very effective and appropriate). Follow-up generalization session procedures were identical to TMCR phase generalization session procedures.

### Data Collection

Sessions were transcribed and coded from video following each session using Systematic Analysis of Language Transcripts (SALT) software, Version 20 (Miller & Iglesias, 2020). Transcription and coding were performed by native Spanish speakers who were unaware of condition changes to mitigate potential bias (Ledford et al., 2018). These transcribers and coders were undergraduate students or bachelor’s or master’s level research staff who had been trained to transcribe and code similar interactions using videos from *EMT en Español* projects. Coded segments were 10 min in length and included 8 min of play, 1 min of routines, and 1 min of book reading from caregiver-child interactions (i.e., in TMCR sessions, the Coach segment).

Dependent variable definitions are in Table 1. Caregiver variables were the caregivers’ use of *EMT en Español Para Autismo* strategies. Target level language for the current study was based on a three-level framework for Spanish language targets developed and used in an ongoing study with LSS children with developmental language disorders (Kaiser & Peredo, 2019–2024). Children in the current study were in the first level; target level and proximal target level language models are described in Table 1. Child dependent variables were the number of total words (NTW), the number of different words (NDW), and the number of times the child communicated with a vocalization, gesture, or word in any mode. All words used by the children were in Spanish during these sessions; however, any words used in English would also have been counted in NTW and NDW.

#### Fidelity and Reliability

Procedural fidelity refers to the extent to which each experimental condition was executed as planned (Barton et al., 2018b). For each type of session (baseline, therapist-child intervention, TMCR, or generalization), 33% of sessions were randomly selected (using the RAND() function in Excel) for procedural fidelity measurement by a trained research team member who did not participate in carrying out sessions. Fidelity checklists specific to each session type were completed from video by a trained observer (other than the interventionist) in a REDCap database (Harris et al., 2009). The interventionists were unaware of which sessions were randomly selected for procedural fidelity measurement. Procedural fidelity averaged 90.2% (75.0–100.0%) across 39 sessions.

Point-by-point interobserver reliability was measured for a randomly selected sample of 33% of sessions for caregiver-child interaction data. The first author performed the random selection of sessions using Excel. Coders were unaware of which sessions were randomly selected for interobserver reliability until after primary transcription and coding of the session were complete. Interobserver reliability for 29 caregiver-child interactions averaged 89.1% (77.5–95.5%) for caregiver data and 87.2% (73.1–95.1%) for child data.

There were concerns regarding low interobserver reliability for some sessions, especially at the beginning of the study. Many disagreements were related to determining whether child vocalizations had communicative intent and whether the adult gave the child enough time to respond. Coding error patterns were reviewed, discussed, and consensus coded at weekly meetings throughout the study (Yoder et al., 2018). Consensus codes were revised in the primary data. Midway through the study, to ensure consistency of coding over the course of the study, a trained coder reviewed and verified coding of sessions that had been transcribed and coded up to that point. Sixty-four caregiver-child interactions (out of 82 coded sessions, 78%) were verified.

### Data Analysis

Caregiver data were graphed and visually analyzed to inform decision-making and to determine the presence or absence of a functional relation for each dyad (Barton et al., 2018a; Gast et al., 2018). Graphs were produced using GraphPad Prism 10 for Windows version 10.1.0 (GraphPad Software, LLC, 2023). The first, second, and fourth authors reviewed primary data weekly throughout the study; decisions were made by consensus. Secondary dependent variables (i.e., generalization and maintenance of caregiver strategy use, child communication) were also graphed and visually analyzed at the end of the study but were not considered in decisions related to phase changes. In addition to visual analysis, we measured the magnitude of change for each demonstration of effect by calculating the log response ratio (LRR) effect sizes. LRRs are advantageous because of the relative insensitivity to procedural variables and the interpretation as percentage of change over baseline (Pustejovsky, 2018, 2019). LRRs were calculated using RStudio version 4.0.2 (R Core Team, 2020) and the batch_calc_es() function in the SingleCaseES package (Pustejovsky et al., 2021). To analyze the social validity of the intervention, responses and notes relevant to the fourth research question (pertaining to how caregivers perceived the intervention approach) from mini-interviews and exit interviews were synthesized by the first author and reviewed by the second and fourth authors. Responses to Likert-type questions were averaged, and family comments were summarized.

## Results

### Caregiver Strategy Use

**Fig. 2:**
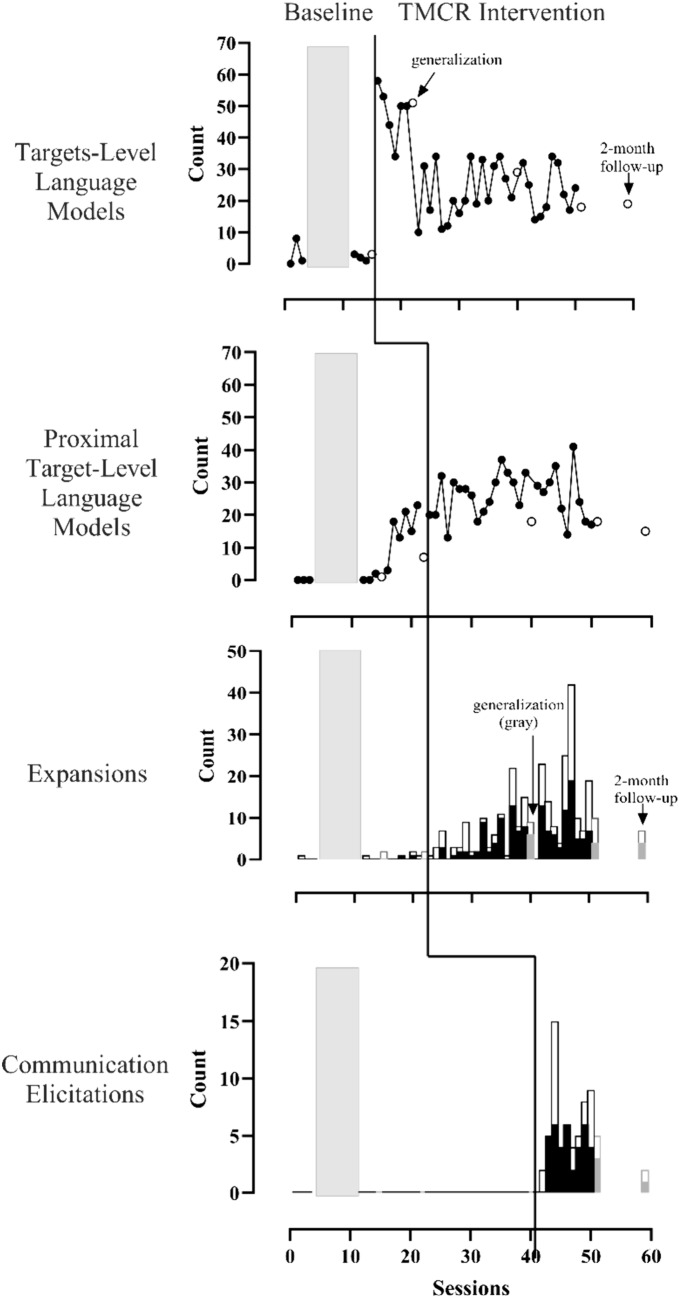
Graphs with four tiers depicting Dayana’s use of strategies. Strategy use increased intervention was introduced for target language and communication elicitations. Proximal targets increased when Tier 1 intervention began. Expansions increased gradually when Tier 2 intervention began. The vertical lines indicate when intervention began for each strategy. Gray boxes indicate when therapist-child intervention occurred. Line graphs show the number of times the caregiver used the targets or proximal targets in coached interactions (black circles) and uncoached interactions (white circles). White bars indicate opportunities to expand or communication elicitation attempts. Black bars indicate expansions or high-quality communication elicitations

Dayana’s data are in Fig. 2. Her use of target level language (Tier 1), expansions (a Tier 2 dependent variable), and communication elicitations (Tier 3) were low and stable during baseline. Contingent target language and communication elicitations immediately increased (within 3 sessions) and her expansions began on a clear increasing trend after the strategies were introduced. In baseline, contingent *proximal* target language (a Tier 2 dependent variable) increased from near zero to approximately 20 (*M* = 15.5, range 3–23) when Tier 1 strategies were introduced. Proximal targets increased again slightly and became more variable (*M* = 26.0, range 13–41) in Tier 2. Dayana generalized her use of all strategies to sessions without coaching during the study and at follow-up, although communication elicitations decreased at the 2-month follow-up. Overall, Dayana increased use of contingent targets by 883% over baseline (*LRRi* = 2.30) and her use of proximal targets by 211% over baseline (*LRRi* = 1.13) with the TMCR intervention. Effect sizes for expansions and communication elicitations were not interpretable because caregiver use of these strategies was near 0 in baseline.

**Fig. 3:**
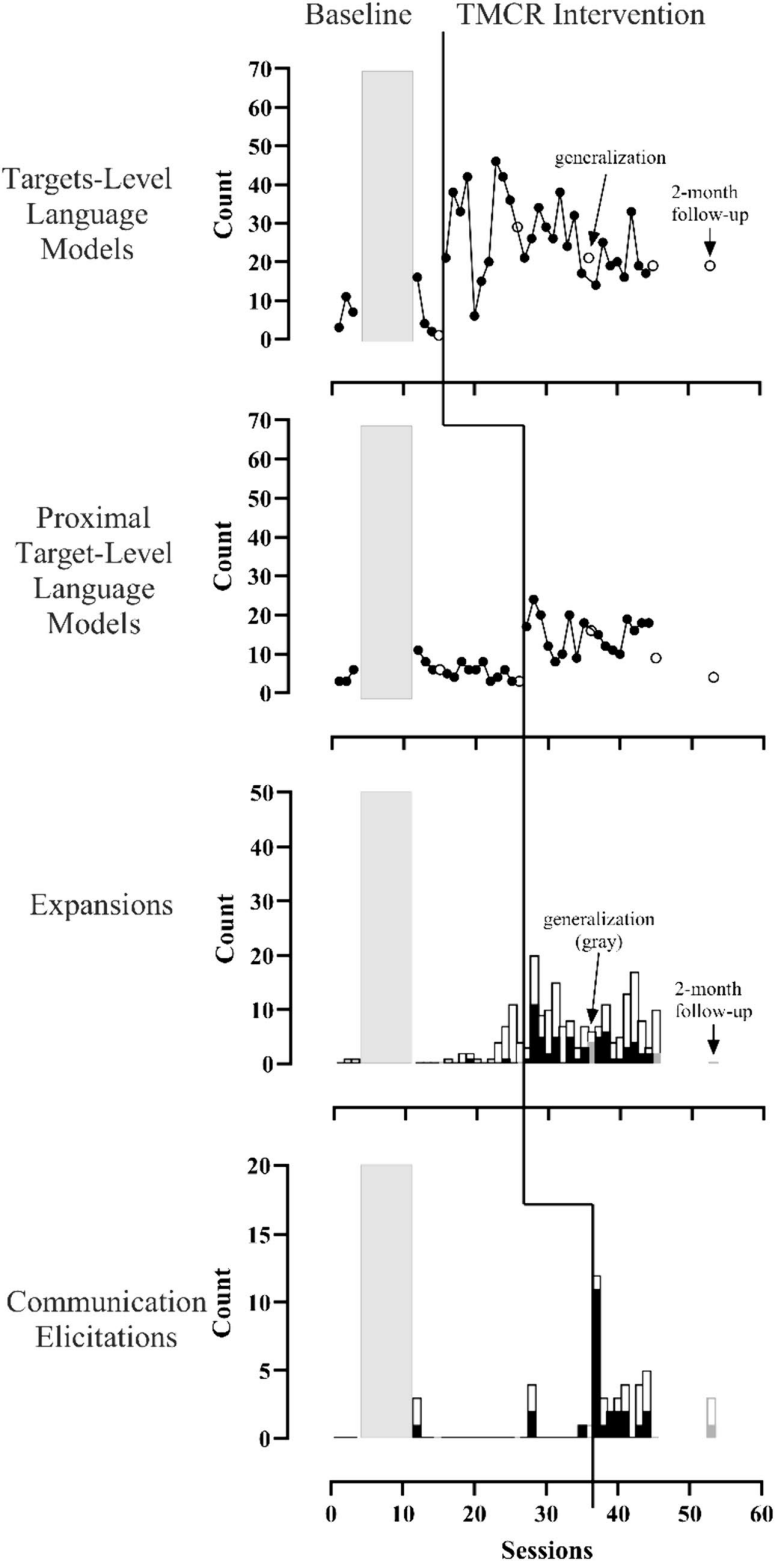
Graphs with four tiers depicting María’s use of strategies. Strategy use increased for each set when intervention was introduced, but contingent targets remained variable. The vertical lines indicate when intervention began for each strategy. Gray boxes indicate when therapist-child intervention occurred. Line graphs show the number of times the caregiver used the targets or proximal targets in coached interactions (black circles) and uncoached interactions (white circles). White bars indicate opportunities to expand or communication elicitation attempts. Black bars indicate expansions or high-quality communication elicitations

María’s data are in Fig. 3. Her use of Tier 2 strategies (proximal target language modeling and expansions) were low and stable during baseline. Contingent target language (Tier 1) and communication elicitations (Tier 3) were somewhat variable during baseline. Data for all strategies demonstrated clear increases in level in the first or second session after the strategies were introduced. There were slight decreasing trends for target language (Tier 1), expansions (a Tier 2 dependent variable), and communication elicitations (Tier 3). Contingent target language remained variable during the intervention phase (*M* = 26.3, range 6–46) but was higher than baseline (*M* = 7.2, range 2–16), on average. Caregiver 2’s generalization to sessions without coaching was variable across strategies. She used targets and communication elicitations but not proximal targets at the follow-up session (there were no opportunities for expansions). Overall, María increased her use of targets by 250% over baseline (*LRRi* = 1.25) and her use of proximal targets by 168% over baseline (*LRRi* = 0.99) with the TMCR intervention. The effect sizes for expansions and communication elicitations were not interpretable because caregiver use of these strategies was near 0 in baseline.

### Child Communication

**Fig. 4:**
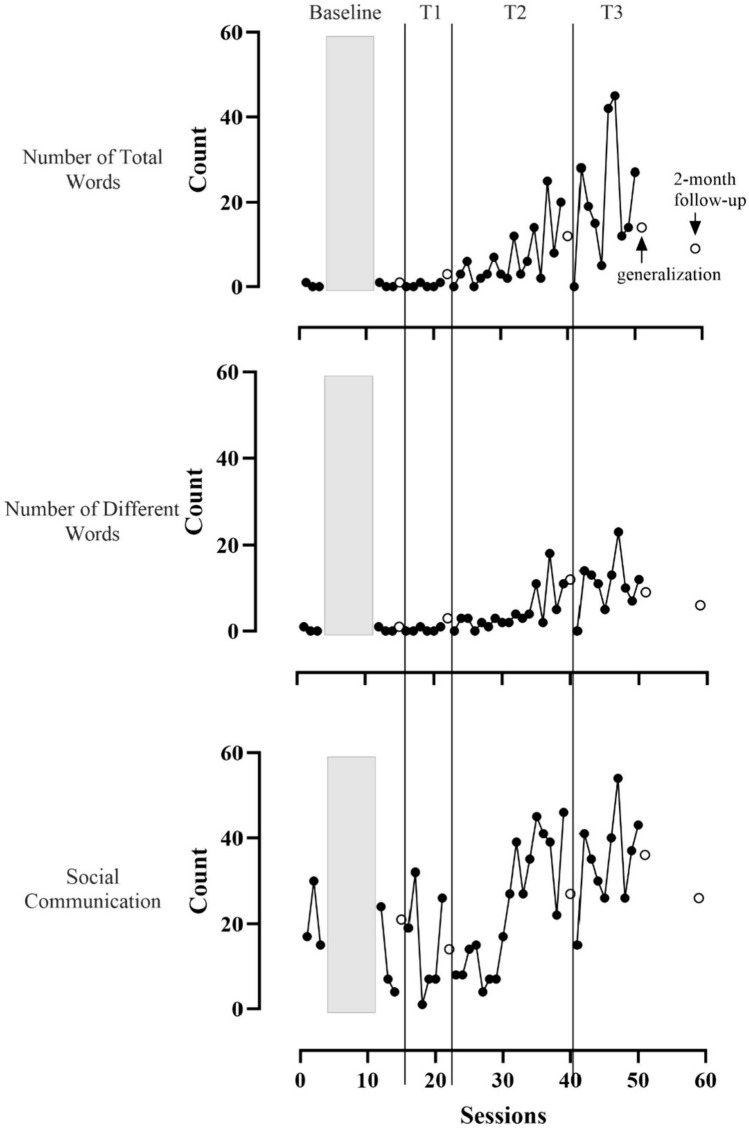
Line graphs showing Daniel’s NTW, NDW, and social communication per caregiver-child interaction in TMCR (black circles) and generalization (white circles) sessions. Vertical lines indicate when new intervention strategies were introduced. NTW and NDW were low in baseline and Tier 1. They increased and became variable in Tiers 2 and 3. Social communication was highly variable and increasing in Tiers 2 and 3. Gray boxes indicate when therapist-child intervention occurred

**Fig. 5:**
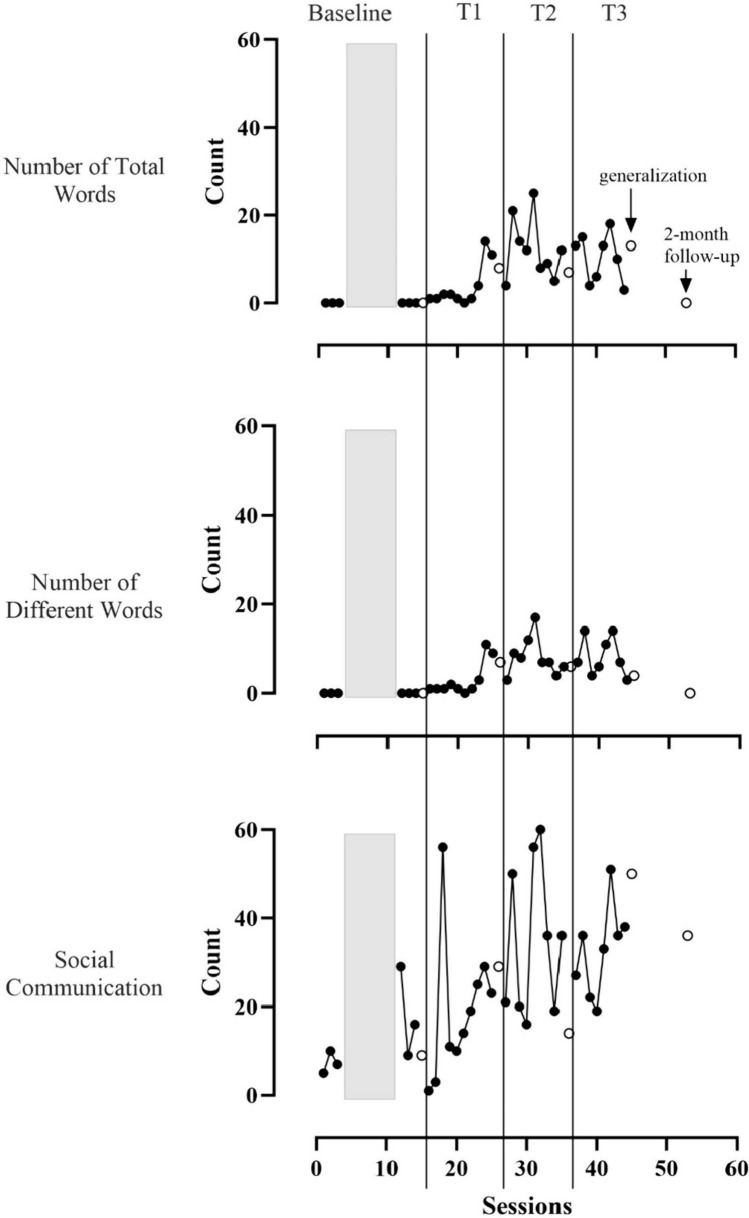
Line graphs showing Luis’s NTW, NDW, and social communication per caregiver-child interaction in TMCR (black circles) and generalization (white circles) sessions. Vertical lines indicate when new intervention strategies were introduced. NTW and NDW were low in baseline. They increased and were variable in all three tiers of intervention. Social communication was highly variable and had an increasing trend across all phases. Gray boxes indicate when therapist-child intervention occurred

Child communication outcomes are displayed in Figs. 4 and 5.

Nearly all words children used were communicated via the SGD. Daniel’s communication with words remained near zero until Tier 2 of intervention, then NTW and NDW increased and became more variable. In Tier 3, Daniel averaged 20.7 total words (range 0–45) and 10.8 different words (range 0–23) per session. For social communication (i.e., utterances with vocalizations, gestures, or words), there was a decreasing trend in baseline (*M* = 16.2, range 4–30), and data were variable through the middle of Tier 2. In Tier 3, Daniel was communicating more frequently on average (*M* = 34.7, range 15–54) than in baseline with a large amount of overlap. Luis communicated using fewer than five words per session until the end of Tier 1 when he used 14 words in one session. NTW and NDW were variable but higher than baseline throughout Tiers 2 and 3 (NTW, *M* = 11.3, range 3–25; NDW, *M* = 8.2, range 3–17). The number of social communication acts was variable throughout the study with an increasing trend. Luis’s social communication in Tier 3 (*M* = 32.8, range 19–51) was higher than in baseline (*M* = 12.7, range 5–29) with some overlap. Notably, Luis’s NTW and NDW decreased to 0 at the follow-up session. Upon arrival, it was discovered that his SGD had been malfunctioning for some time. It was repaired prior to the follow-up generalization session.

### Social Validity

At the exit interview, caregivers reported that the most helpful component of TMCR was watching the interventionist model the intervention with the child (Dayana) or practicing implementing the strategies with coach feedback (María). Both reported using *EMT en Español Para Autismo* strategies every day, including during play, pre-academic activities (e.g., coloring, book-sharing), and caregiving routines (e.g., bath time, mealtime). They rarely used intervention strategies during housekeeping routines (e.g., laundry, cleaning). Both participating caregivers taught the strategies to other family members. When asked how the intervention could be improved, one family suggested adding music to some of the activities to help the child concentrate. The other family suggested a longer intervention period. Dayana rated all strategies on which she was trained with a 5 (very effective and appropriate). María rated all strategies with a 4 or 5 except for the Tier 1 strategies of reducing instructions and questions, which she rated a 1 (ineffective and inappropriate). Both families reported difficulty with managing the SGD. María did not agree with allowing children to frequently use tablets and phones, but she could see her child was happy when he was understood by others. Daniel’s family became frustrated when he became so focused on his device that he did not participate in the activity at hand (e.g., eating his food at mealtime).

## Discussion

The primary purpose of the current pilot study was to test the effects of the collaborative TMCR approach to teach *EMT en Español Para Autismo* strategies to two Latina Spanish-speaking caregivers with their toddlers on the autism spectrum. Social validity analyses indicated both families felt the intervention was effective with some concerns related to use of the SGDs. This study extends the small research base on culturally and linguistically adapted early communication interventions for LSS families and their children on the autism spectrum.

The study’s development and design had unique strengths. First, the intervention was initially adapted for LSS families of young children with language delays (Peredo et al., 2018, 2022) and adapted again for children on the autism spectrum. Second, the intervention was individualized for each participating dyad based on repeated family interviews throughout the study and a direct therapist-delivery phase of intervention prior to caregiver coaching. The essential components of EMT that support children’s language development (e.g., environmental arrangement, contingent language modeling) were maintained; however, these components allowed the therapist to build and maintain rapport with the family during baseline and while teaching the intervention strategies. They also supported collaboration and family preference related to intervention materials, activities, engagement supports, and introduction and programming of the SGD.

### TMCR and EMT en Español Para Autismo Strategies

There was a clear functional relation between systematic implementation of the TMCR approach and use of *EMT en Español Para Autismo* strategies for one of the caregivers (María). In other words, she increased her use of specific *EMT en Español Para Autismo* strategies when and only when she was taught each strategy using the TMCR approach (Gast et al., 2018). For Dayana, there were three demonstrations of the effect of TMCR on use of target level language, expansions, and communication elicitations (Gast et al., 2018). However, the confidence in the functional relation was weakened by the covariation between contingent target and proximal target level language. The increase in proximal target level language (a Tier 2 strategy) corresponded with the introduction of Tier 1 strategies. This covariation indicates that use of proximal and target level phrases were not fully independent behaviors for Dayana; rather, she began using simpler phrases at a higher rate when target level language was introduced and did not discriminate targets from proximal targets. Although unexpected, this response generalization is not surprising given the precise linguistic distinctions between target-level and proximal target-level language targets as shown in Table 1. For example, the label for popsicle would be a target if it were in singular form (la paleta) and a proximal target if it were in plural form (las paletas). Many caregivers would likely benefit from being taught target level and proximal target level language simultaneously rather than teaching proximal targets at the same time as expansions.

The caregivers reported the TMCR approach to be effective and helpful for them in learning the strategies. They reported that most of the *EMT en Español Para Autismo* strategies were effective and appropriate with one exception. María indicated that reducing instructions and questions to balance matched turns was ineffective and inappropriate for her in interactions with her child. This finding is somewhat consistent with other *EMT en Español* studies in which caregivers reported a cultural tension with reducing questions and directions but found it to be an effective strategy for their child (Peredo et al., 2018, 2020). Further research on the perceived effectiveness of reducing test questions and behavioral directions from LSS caregivers of children on the autism spectrum will help determine if further adaptation of this strategy is needed.

The findings should be interpreted in light of the fact that caregiver opportunities to practice and demonstrate skills such as use of targets and expansions were contingent on the opportunities presented by child communication and engagement. Simply put, for caregivers to immediately increase their behavior, there had to be child-presented opportunities to respond. Measuring contingent behavior in this way closely reflects the posited active ingredient of the intervention (Dillehay, 2023), and it may explain differences between results in the current and previous studies. Unlike in the Peredo et al. (2018) study, in the current pilot study, Dayana’s use of expansions increased gradually. Daniel often activated the same word many times in a row, and it was difficult to determine his communicative intent. This could have influenced Dayana’s ability to respond contingently using expansions and coders’ interobserver agreement.

### Independent Use of Strategies

Use of strategies generalized or partially generalized to sessions without coaching support, including at follow-up. The overall number of generalization sessions was small, and the context only differed from the intervention context by one variable (the absence of coaching support); however, the current data are encouraging when interpreted alongside the caregiver reports that they used *EMT en Español Para Autismo* strategies throughout the week during play, book reading, and routines. The individualization of the intervention (i.e., collaborative selection of toys, interviews) may also have supported generalization by ensuring intervention activities aligned with family activities outside the study and that multiple family members were involved (DuBay et al., 2018). María’s use of Tier 2 strategies did not generalize or maintain at the 2-month follow-up. At that session, she did not have opportunities to expand because Luis did not use any verbal utterances. Luis’s decrease in verbal communication may have been related to lack of access to his SGD prior to the follow-up session. Families would likely benefit from booster sessions and check-ins for technical support for long-term generalization and maintenance of strategy use (Kent-Walsh & McNaughton, 2005).

### Child Outcomes

While the design of this pilot study did not control for possible effects of maturation on child communication, both children in the study demonstrated significant growth during the 6–7 months they were in the study. Neither child was receiving any other targeted language intervention in Spanish at the time that might have accounted for the growth. Both children began using SGDs provided during the study (and were not using them during baseline), which was likely critical for supporting their increased communication, in addition to implementation of the *EMT en Español Para Autismo* strategies with aided modeling by their caregivers and the therapists. Also important to note regarding child outcome data was that, to increase coding reliability, a decision was made to score child vocalizations or activation of SGD symbols as communicative if the caregiver responded contingently. Therefore, it is possible that a greater proportion of child vocalizations and SGD activations were coded as communicative in later sessions than in earlier sessions, reflecting both increases in caregiver responsiveness and differences in child communication.

### Limitations

The first major limitation to this pilot study was the number of participants. Four families enrolled in the study, and only two families completed the intervention. Both families that dropped out indicated that they did not want to tire their child by having them in too many therapies. This speaks to the time and effort that families must contribute to participating in an intensive early childhood intervention and particularly to the research requirements associated with added paperwork and scheduling of sessions. For researchers, it is important to consider shorter baselines, limited paperwork, and designs that require fewer sessions. Solutions in practice may include a greater degree of collaboration between the multiple providers (Part C developmental therapists, speech language pathologists, and others), more efficient use of therapy time, and continuously engaging with families to understand their priorities in choosing services and delivery models.

Another limitation was that introduction of new *EMT en Español Para Autismo* strategies roughly coincided with minor changes to routine contexts for intervention (described in the Participants section). After mini-interviews, routines began to include coloring for Daniel and brushing teeth and washing dishes for Luis. It is possible that the new contexts influenced the caregivers’ use of strategies at the time they were introduced; however, those changes would likely have affected caregivers’ data in all tiers. Routine contexts also comprised a small proportion of the data collection period in each session (1 min out of 10 min).

Other limitations pertained to interpretation of child outcomes. Given the study design, child communication outcomes could not be attributed specifically to caregiver use of *EMT en Español Para Autismo* strategies. The children received the full intervention from the therapist during the initial direct intervention phase and during the model portions of the TMCR sessions. The caregivers were not taught the full intervention until Tier 3 near the end of the study. Additionally, the contribution of the children’s access to the SGD could not be separated from the effects of the *EMT en Español Para Autismo* intervention delivered by the therapist and the caregiver. Future studies should investigate caregiver implementation of *EMT en Español Para Autismo* with SGDs using a study design that allows for detection of effects of caregiver training alone on child outcomes.

### Future Directions for Research

Future research should build on the current findings by systematically replicating the current study with additional LSS families from diverse backgrounds. Children on the autism spectrum are heterogeneous as well, with different interests and abilities including social communication, receptive and expressive language skills, and engagement in play-based activities (McDuffie et al., 2012). Systematic assessment, the direct therapist intervention component, as well as the collaborative interview process and strategic individualization in this pilot study present one potential model for future studies to individualize *EMT en Español Para Autismo* for diverse LSS participants.

Future research should also expand the intervention to address all aspects of using AAC with this population of families. Although both children demonstrated increases in verbal communication using SGDs, one caregiver indicated that she was reluctant to use it at the beginning of the intervention and the other family reported difficulty managing the SGD during everyday routines. Researchers should continue to develop materials and methods for teaching LSS caregivers about AAC (De Leon et al., 2023), the evidence to support its use by children on the autism spectrum (e.g., Hampton et al., 2020), and instruction in how to model language using SGDs (Biggs et al., 2018; Sevcik et al., 1995). Low-tech forms of AAC may also be effective and preferred by some families. These materials should be culturally and linguistically adapted with the help of LSS families, as were the workshops for the current study. Future studies should also delineate systematic procedures for selection of Spanish and English vocabulary to include on the devices, incorporating principles of typical bilingual Spanish and English language development and individualized family communication needs (Binger et al., 2023; Soto & Cooper, 2021).

### Implications for Practice

Practitioners could apply the collaborative interview process when working with LSS families and children on the autism spectrum using the protocols in the Online Resource and published by Peredo (2016). Conversations or interviews prior to implementing family-centered intervention have been recommended when working with culturally and linguistically diverse families (Cycyk & Iglesias, 2015; Peredo, 2016). In the current study, these interviews systematically occurred at regular intervals throughout intervention. Intervention should ideally be provided by practitioners that speak Spanish when that is the family’s home language. However, practitioners working with interpreters or with limited proficiency in the family’s home language could also use similar interview questions to structure conversations to better understand family values, frequent activities, and preferences.

Bilingual practitioners may also consider a direct intervention component when working with LSS families with toddlers on the autism spectrum. A direct therapist intervention phase prior to caregiver coaching could support planning and collaboration by giving the practitioner a better understanding of potentially needed supports (e.g., AAC, behavior supports). A continued direct intervention throughout the caregiver coaching phase, either via the Model component of TMCR or additional direct intervention sessions, could support overall dosage of intervention received by the child. This dual implementer approach could ease the pressure on caregivers to deliver the entire dosage of intervention necessary to see language skill gains while still engaging and empowering families to support their child’s growth.

## Conclusion

Few intervention studies have focused specifically on the experiences, needs, and preferences of LSS families with children on the autism spectrum. This study demonstrated effective application of the TMCR approach to teach caregivers a culturally, linguistically, and individually adapted intervention. The caregivers in the current pilot study implemented *EMT en Español Para Autismo* strategies with their children on the autism spectrum, generalized use of most of the strategies to unsupported interactions, and gave positive feedback about their experience with the intervention. The children increased the frequency and diversity of communication with their caregivers over time. This study contributes to the literature on family-centered naturalistic developmental behavioral interventions for diverse families and children on the autism spectrum. More systematic inquiry is needed to understand the effects and social validity of the TMCR approach and *EMT en Español Para Autismo* strategies for diverse families.
